# McKeown Esophagectomy for Esophageal Cancer Following Pancreaticoduodenectomy Using a Mesenteric Approach for Pancreatic Cancer: A Case Report

**DOI:** 10.70352/scrj.cr.25-0204

**Published:** 2025-09-03

**Authors:** Takeshi Miwa, Tomoyuki Okumura, Yoshihisa Numata, Mina Fukasawa, Nana Kimura, Toru Watanabe, Katsuhisa Hirano, Isaya Hashimoto, Kazuto Shibuya, Isaku Yoshioka, Satoshi Onoda, Toshihiko Satake, Tsutomu Fujii

**Affiliations:** 1 Department of Surgery and Science, Faculty of Medicine, Academic Assembly, University of Toyama, Toyama, Toyama, Japan; 2 Department of Plastic and Reconstructive Surgery, Faculty of Medicine, Academic Assembly, University of Toyama, Toyama, Toyama, Japan

**Keywords:** McKeown esophagectomy, esophageal cancer, colon reconstruction, pancreaticoduodenectomy, mesenteric approach

## Abstract

**INTRODUCTION:**

There are no reports of patients undergoing McKeown esophagectomy for esophageal cancer after undergoing pancreaticoduodenectomy for pancreatic cancer. We report the case of a patient who underwent subtotal esophagectomy and colon reconstruction after pancreaticoduodenectomy using the mesenteric approach.

**CASE PRESENTATION:**

A 71-year-old male was diagnosed with advanced esophageal cancer. Four years prior to diagnosis, he underwent subtotal stomach-preserving pancreaticoduodenectomy using the mesenteric approach for pancreatic surgery, followed by Child’s reconstruction surgery. After undergoing 3 cycles of neoadjuvant chemotherapy with docetaxel, cisplatin, and 5-fluorouracil, the patient was scheduled for a subtotal esophagectomy. The middle colic artery was transected using the mesenteric approach, and the upper jejunum was utilized for Child’s reconstruction surgery. A 2-stage procedure involving McKeown esophagectomy and left-sided colon reconstruction was planned. The 1st stage of the procedure involved robot-assisted subtotal esophagostomy in the prone position, followed by cervical esophagostomy and gastrostomy. The patient underwent the 2nd stage of the surgery after approximately 1 month of parenteral nutrition via a gastrostomy tube. The transverse colon was mobilized and transected at the hepatic flexure. The left side of the mesocolon, which is fed by the left colic artery, was then pulled up through the antethoracic route. The right internal thoracic artery and vein were anastomosed to the marginal artery and vein of the transverse colon, respectively, for supercharge and superdrainage. Reconstruction involved esophago-colonic and colonic–gastric anastomoses. The patient was discharged without postoperative complications, and no signs of recurrence were observed at the 2-year postoperative follow-up.

**CONCLUSIONS:**

Subtotal esophagectomy for esophageal cancer after subtotal stomach-preserving pancreaticoduodenectomy using a mesenteric approach and colon reconstruction can be safely performed in 2 stages. The optimization of pancreaticoduodenectomy for pancreatic cancer could improve the long-term survival of patients with 2nd primary esophageal cancer, for which radical esophagectomy is necessary.

## Abbreviations

DCF
: docetaxel/cisplatin/5-FU
ICG
: indocyanine green
ITA/V
: internal thoracic artery and vein
LCA
: left colic artery
MCA
: middle colic artery
PD
: pancreaticoduodenectomy
SSPPD
: subtotal stomach-preserving pancreaticoduodenectomy

## INTRODUCTION

Esophageal cancer is a malignant neoplasm with a poor prognosis, ranking as the 6th most common cause of death worldwide (5.3%).^1)^ The incidence of esophageal cancer is relatively high in East Asia. In Japan, squamous cell carcinoma accounts for 90% of esophageal cancers.^2)^ The standard treatment for advanced esophageal squamous cell carcinoma in Japan is neoadjuvant chemotherapy followed by radical esophagectomy.^3)^ Esophagectomy is a highly invasive surgery, and the reconstruction method should be selected based on the previous surgery and the location of the tumor. The gastric tube is usually used for reconstruction, but, in some cases, other organs may be used for reconstruction after gastrectomy. In this case, we performed radical subtotal esophagectomy and 2-stage colon reconstruction for a patient who was diagnosed with middle thoracic esophageal cancer after SSPPD using a mesenteric approach for pancreatic head cancer.

## CASE PRESENTATION

**Fig. 1:**
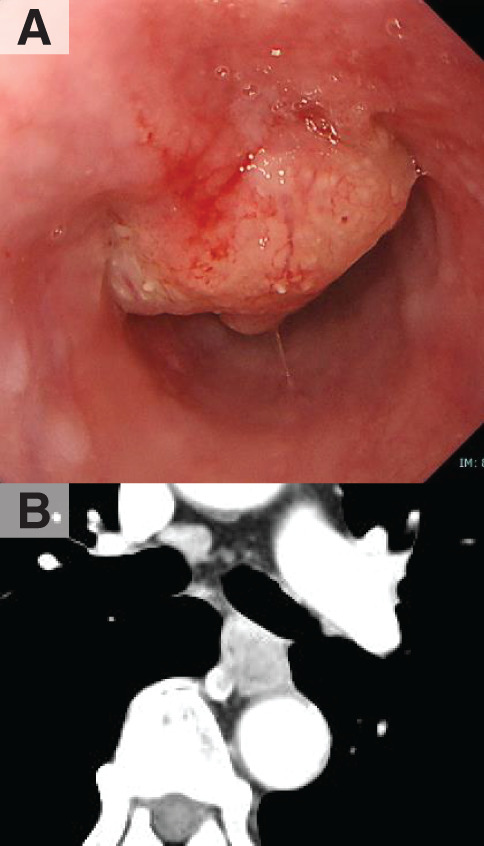
Findings of upper gastric endoscopy and CT. Upper gastric endoscopy revealed a type 1 tumor located in the middle thoracic esophagus (**A**), and contrast-enhanced CT revealed thickening of the wall of the esophageal tumor (**B**).

A 71-year-old man with a 2-month history of dysphagia visited our clinic. Four years prior, he underwent SSPPD using a mesenteric approach for pancreatic head adenocarcinoma (pT3, pN0, pM0, pStage IIa, R0) and Child’s reconstruction at our hospital.^4)^ He received adjuvant chemotherapy with S-1 (100 mg/day), was followed up, and showed no signs of pancreatic cancer recurrence. Endoscopy at our hospital revealed a type 1 tumor in the middle thoracic esophagus, which was diagnosed as squamous cell carcinoma upon biopsy (**Fig. 1A**). A contrast-enhanced CT scan revealed a thickened esophageal wall with an enlarged lymph node under the tracheal bifurcation (**Fig. 1B**). PET revealed an accumulation of fluorodeoxyglucose in the primary tumor and mediastinal lymph node, but no other abnormal accumulations, which suggested distant metastasis and the recurrence of pancreatic cancer. We diagnosed the patient as having middle thoracic advanced esophageal cancer (cT3, cN1, cM0, cStage III)^5)^ and planned surgery after neoadjuvant chemotherapy with 3 courses of DCF.^6)^ Although we decreased the dose of the 3rd course of DCF due to oral mucositis, the patient completed all 3 courses of chemotherapy, and the tumor appeared to have decreased in size, indicating a partial response. Then, radical esophagectomy and reconstruction were planned.

**Fig. 2:**
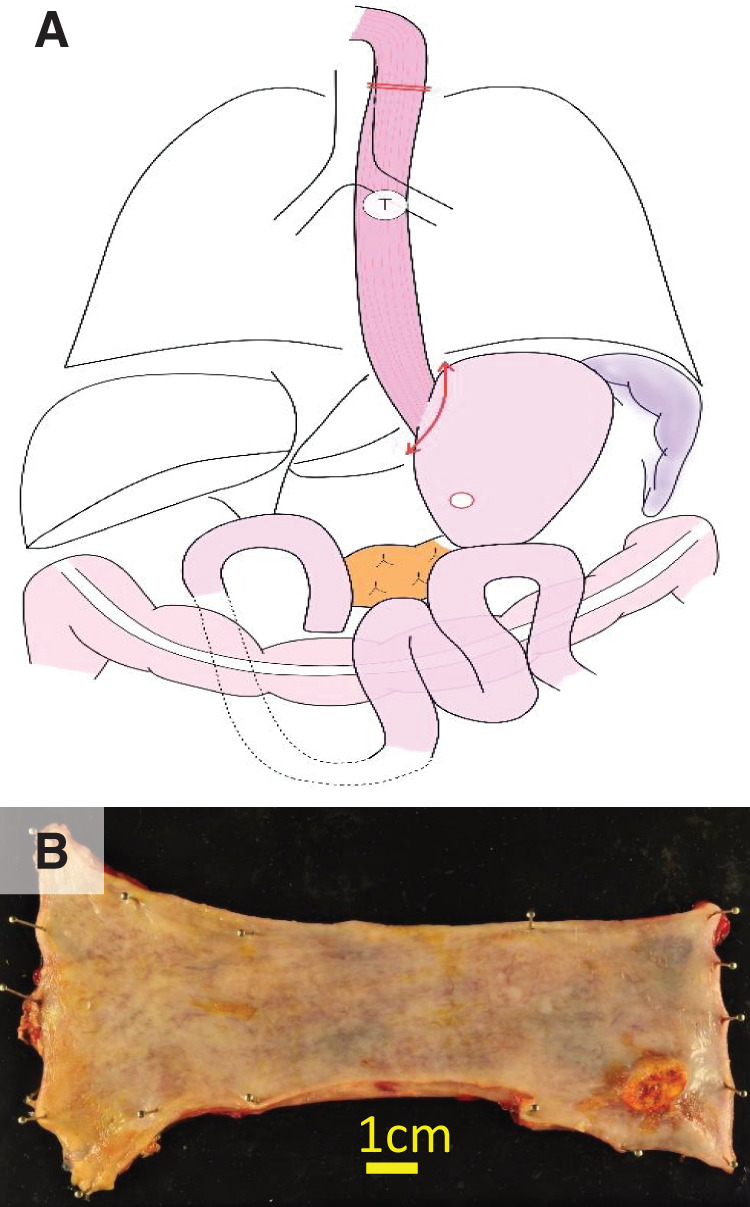
The 1st-stage subtotal esophagectomy. Robot-assisted subtotal esophagectomy in the prone position, followed by cervical esophagostomy and gastrostomy (**A**) and resection of a specimen of the esophagus (**B**). T, tumor

**Fig. 3:**
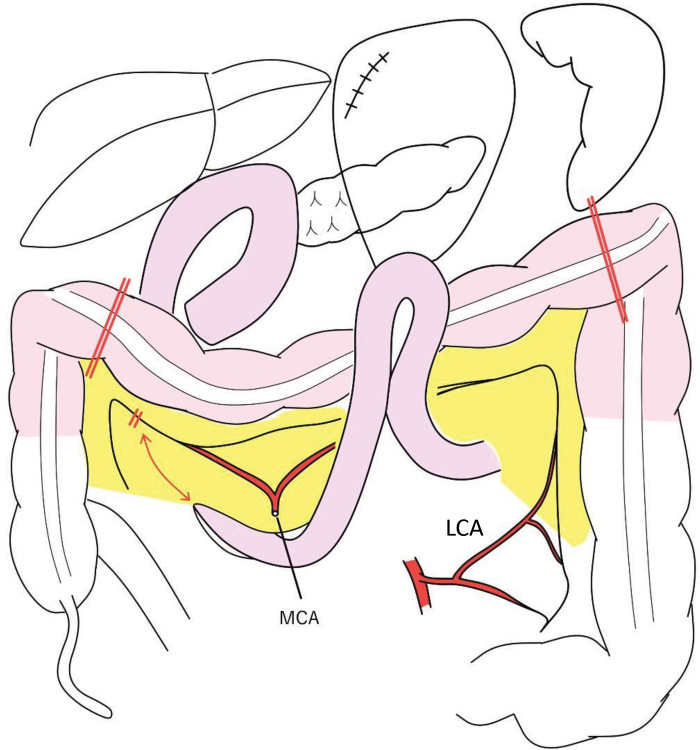
The 2nd-stage reconstruction surgery. The transverse colon was mobilized and transected at the hepatic fold while the pedicled LCA was feeding. The MCA was transected during pancreaticoduodenectomy with the mesenteric approaCh. LCA, left colic artery; MCA, middle colic artery

**Fig. 4:**
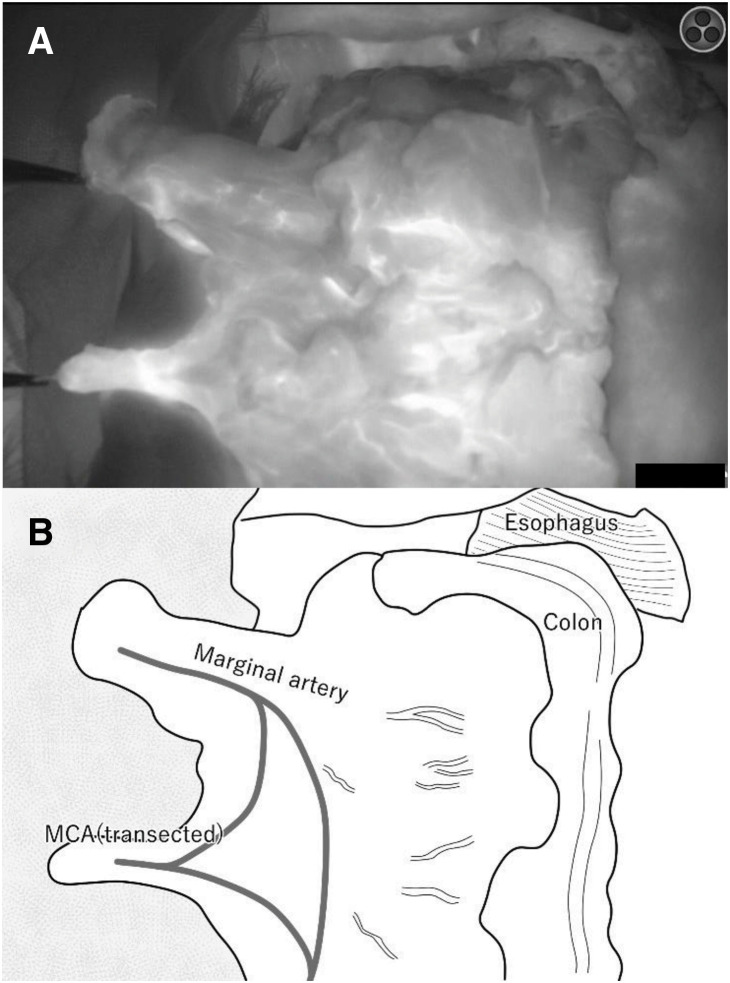
ICG fluorescence imaging. ICG imaging (**A**) and schematic (**B**) of the pulled-up transverse colon after an injection of 2.5 mg of ICG. The marginal artery of the colon and the MCA had good blood flow after transection according to ICG imaging. ICG, indocyanine green; MCA, middle colic artery

**Fig. 5:**
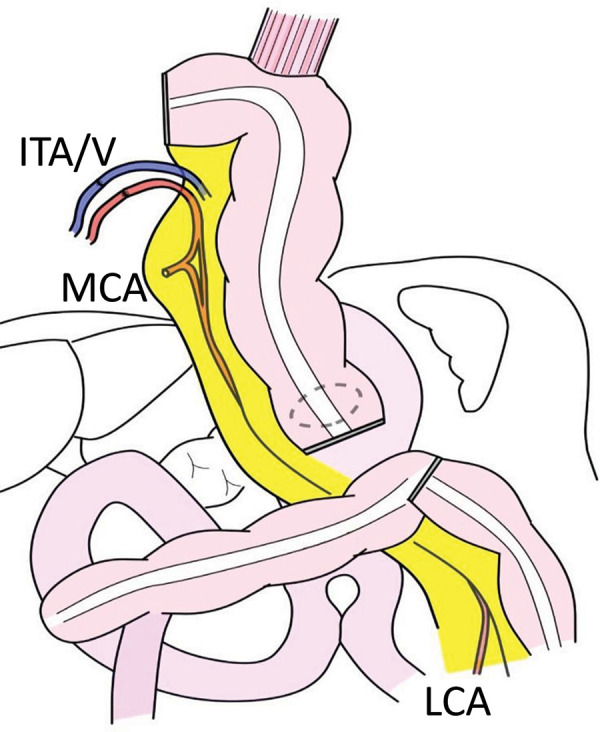
Schematic of pedicled colonic reconstruction. The esophago-colostomy and colo-gastrostomy were performed while the LCA was feeding. Supercharge and superdrainage via the right ITA/V to the marginal colonic vessels were added. ITA/V, internal thoracic artery and vein; LCA, left colic artery; MCA, middle colic artery

In the previous SSPPD using a mesenteric approach, the right gastric artery, right gastroepiploic artery, 1st jejunal artery, and MCA were transected, and the jejunum in the 2nd and 3rd jejunal artery regions was used for Child’s reconstruction. Gastric reconstruction after SSPPD was impossible, and ileocolic (right-sided colonic) reconstruction was not suitable because the MCA had been transected. Moreover, pedicled jejunal reconstruction was considered difficult because the jejunum in the 4th and lower jejunal artery regions was not expected to reach the cervical esophagus. Therefore, we planned left-sided colon reconstruction with the pedicled transverse colon feeding from the LCA. Because it is a highly invasive procedure, we decided to perform it in 2 stages. In the 1st stage of surgery, a robot-assisted subtotal esophagectomy with mediastinal lymph node dissection was performed in the prone position. Subsequently, through an upper abdominal laparotomy, the pericardial lymph node dissection, transection of the stomach just below the cardia using a linear stapler, and tube gastrostomy were carried out. The upper esophagus was transected proximally to the tumor, and a cervical esophagostomy was performed (**Fig. 2**). The operation time and blood loss were 528 min and 100 g. Enteral nutrition was started postoperatively, the patient was temporarily discharged on POD 15, and reconstructive surgery was scheduled 29 days after esophagectomy. In the 2nd stage of surgery under laparotomy in the supine position, the transverse colon and the mesentery were mobilized after careful dissection of adhesions formed following SSPPD (**Fig. 3**). The left and right sides of the colon were also mobilized for reconstruction. The marginal vessels at the hepatic fold of the transverse colon were clamped, and blood flow through the transverse colon from the LCA was confirmed using fluorescence imaging following an intravenous injection of 2.5 mg of ICG. The transverse colon was cut at the hepatic fold, and the oral side of the distal colon was pulled up to the stump of the cervical esophagus via the anterior thoracic wall route. An additional 2.5 mg of ICG was injected intravenously to confirm whether the blood flow of the elevated colon was maintained (**Fig. 4**). The right ITA/V were anastomosed to the marginal artery and vein of the transverse colon by plastic surgeons for supercharge and superdrainage. After dissection of the esophagostomy and removal of the gastrostomy tube, the cervical esophagus and the oral side of the elevated colon were anastomosed. The middle segment of the colon was cut for the colon–stomach anastomosis to avoid damaging the mesocolon, and colon–stomach and right colon–left colon anastomoses completed the reconstruction procedure (**Fig. 5**). A tube jejunostomy was performed for early enteral nutrition. The operation time and blood loss were 860 min and 1505 g. The patient was allowed to start eating jellied foods on POD 8 and was discharged on day 17 without complications. The pathological diagnoses were pT2, pN0, M0, pStage II, R0, and the chemotherapeutic effect was grade 1a. Enteral nutrition was initiated on the 1st POD and continued for 2 months after surgery. Although anastomotic stenosis occurred 3 months after surgery, it was possible to treat it with endoscopic dilation. He was followed up without postoperative chemotherapy, and no recurrence was observed 2 years after surgery.

## DISCUSSION

McKeown esophagectomy is a radical surgical treatment for esophageal cancer and is classified as a highly invasive surgical technique. To determine the optimal reconstruction method following esophagectomy, it is essential to consider the previous surgical intervention. PD is a difficult surgery performed as a radical treatment for pancreatic head cancer and biliary tract cancer and requires the reconstruction of the stomach, pancreas, and bile duct using the upper jejunum. This case required the consideration of resection and reconstruction methods for the radical treatment of esophageal cancer after SSPPD. To our knowledge, there have been 3 reports of esophagectomy after PD. Fukaya et al.^7)^ reported middle-lower esophagectomy with total gastrectomy for long-segment benign esophageal strictures due to gastroesophageal reflux disease after pylorus-preserving PD for lower bile duct carcinoma. They performed transverse colon reconstruction via the internal thoracic route. Asai et al.^8)^ reported a case of upper-to-middle-thoracic esophageal metastasis of breast cancer after SSPPD for ampullary cancer. They performed 2-stage subtotal esophagectomy and reconstruction with a pedicled jejunum via the antethoracic route, allowing supercharge and superdrainage via the right ITA/V. Moriwake et al.^9)^ reported subtotal esophagectomy with free jejunal reconstruction for superficial esophageal cancer after pylorus-preserving PD for intraductal papillary mucinous neoplasm. They used a free jejunal flap fed by the inferior jejunal artery anastomosed to the right internal thoracic artery through the anterior thoracic wall route. Gastric reconstruction was not performed in any cases. In 1 case, the patient was diagnosed with esophageal cancer but did not receive preoperative chemotherapy due to the early stage. In the reported cases, PD was not performed using the mesenteric approach, which requires transection of the MCA. The reason why there are few reported cases of esophagectomy after PD is that (1) PD is usually indicated for the treatment of pancreatic and biliary cancers, which have poor prognosis, and many patients may not survive long-term after PD; and (2) it is difficult to plan reconstruction surgery following esophagectomy after PD. In addition, there is only 1 report of surgical treatment for esophageal cancer after PD. Surgery for esophageal cancer often requires mediastinal lymphadenectomy and perioperative chemotherapy, which makes surgery for esophageal cancer more invasive.

PD is performed for carcinomas with a poor prognosis, including pancreatic cancers and biliary tract cancers. However, recent advances in chemotherapy may increase the number of patients who survive long-term after PD. Pancreatic cancer is a malignancy with a poor prognosis; it is the 7th leading cause (4.5%) of cancer deaths worldwide.^1)^ In Japan, pancreatic cancer is also considered to have a poor prognosis.^10)^ However, recent studies have revealed the efficacy of preoperative chemotherapy with gemcitabine and S-1 for patients with resectable pancreatic cancer.^11)^ Additionally, favorable outcomes of conversion surgery in conjunction with chemoradiotherapy for unresectable pancreatic cancer have been documented.^12)^ Moreover, a mesenteric approach that prioritizes lymph node dissection around the superior mesenteric artery has been proven beneficial and requires the resection of the origin of the MCA.^13,14)^ In this case, advanced esophageal cancer was diagnosed 4 years after PD using a mesenteric approach for pancreatic head cancer. The patient showed no signs of pancreatic cancer recurrence and was able to complete treatment for esophageal cancer. As treatments for pancreatic cancer continue to improve, the number of long-term survivors of pancreatic cancer is expected to increase, and the risk of developing metachronous primary cancer in other organs is also expected to increase.

The standard treatment for advanced esophageal cancer is preoperative chemotherapy, followed by subtotal esophagectomy with mediastinal lymph node dissection.^15)^ For advanced esophageal cancer, surgery alone is associated with a high risk of recurrence. Preoperative chemotherapy has been proven superior to postoperative chemotherapy in Japanese clinical trials.^16)^ In addition, the survival benefit of DCF triplet chemotherapy as a preoperative therapeutic regimen has been demonstrated.^6)^ Surgery alone may be considered for patients who do not tolerate chemotherapy well, but the patient in this case was clinically diagnosed with positive lymph node metastasis, and surgery alone was considered less curative. Definitive chemoradiotherapy is an important treatment option for cases in which curative resection is deemed technically unfeasible or the patient is expected to have poor surgical tolerance.^17)^ In the present case, reconstruction using the colon after prior PD was anticipated to be technically demanding and highly invasive; therefore, the treatment strategy was carefully considered, including the possibility of chemoradiotherapy. We planned to proceed with definitive chemoradiotherapy instead of surgery if there was any deterioration in the patient’s general condition or emergence of factors that might compromise surgical safety during neoadjuvant chemotherapy. Fortunately, the patient experienced minimal adverse events and completed chemotherapy while maintaining good physical status, which allowed us to proceed with surgery. Although the 2nd-stage operation required a prolonged operative time due to the complexity of adhesiolysis and colonic reconstruction, intraoperative hemodynamics were stable, and postoperative recovery was uneventful. Given the invasiveness of reconstructive procedures in such cases, definitive chemoradiotherapy should be fully considered when there are concerns about perioperative risk or overall patient condition.

The stomach (whole stomach or gastric tube), jejunum, and colon are selected for reconstruction after subtotal esophagectomy. The stomach is the most common reconstruction organ, but it cannot be used if the right gastroepiploic artery has been transected. Pedicled jejunal reconstruction with supercharge and superdrainage has been reported as another reconstruction method.^18)^ Normally, the 2nd and 3rd jejunal arteries are cut, and the jejunum is pulled up. In this case, the jejunum, which was used in the reconstruction of the pancreas, had to be preserved, and it was necessary to lift the jejunum and the 4th jejunal artery, which was unlikely to reach the cervical esophagus.^19)^ Ileocolonic (right-sided colon) reconstruction requires the MCA to serve as the feeding artery and is therefore difficult to perform after the mesenteric approach. Therefore, we opted for left-sided colonic (transverse to the descending colon) reconstruction and selected the LCA as the feeding artery in this case. The mesenteric approach requires the origin of the MCA to be cut for lymph node dissection around the superior mesenteric artery, but the left- and right-branch bifurcations are preserved. Thus, the transverse colon was fed by the marginal vessels that flow from the left and right colic arteries, and abundant blood flow from the marginal vessels was expected. The elevated transverse colon appeared to have adequate blood flow on ICG fluorescence images,^20)^ but additional measures to increase blood flow were performed to reduce the risks of postoperative congestion and colon necrosis.

Radical resection of esophageal cancer typically requires abdominal lymph node dissection. In the present case, the patient had previously undergone SSPPD, which posed a risk of ischemic gastropathy if the left gastric artery were resected. Therefore, the left gastric artery was preserved, and abdominal lymph node dissection was limited to the region around the cardia.

The reasons we opted for a 2-stage surgery instead of a highly invasive 1-stage surgery are as follows: The patient was scheduled for transverse colon reconstruction requiring mobilization of the ascending, transverse, and descending colon. The patient had undergone preoperative chemotherapy and required lymph node dissection with esophagectomy. Surgeons should opt for a 2-stage surgery if the combination of these procedures in a 1-stage surgery would be considered highly invasive or high risk.^21)^ The patient received enteral nutrition during the approximately 1-month waiting period between the 2 stages of the surgery, and reconstruction was successfully performed without any complications.

## CONCLUSIONS

In this case, McKeown esophagectomy for a patient who had previously undergone SSPPD by mesenteric approach was successful with 2-stage colonic reconstruction. The development of improved treatment modalities for pancreatic cancer has the potential to increase the number of long-term survivors who could develop secondary primary esophageal cancer.

## DECLARATIONS

### Availability of data and materials

The datasets for this case study can be obtained from the corresponding author upon a reasonable request.
